# Ultrasound‐guided hepatic portal vein injection is not a reproducible technique for delivery of cell therapies to the liver in mice

**DOI:** 10.1111/dme.15192

**Published:** 2023-08-17

**Authors:** Sophie L. Walker, June Noble, Adrian Thomson, Carmel M. Moran, David Mellis, I‐Ning Lee, Lisa J. White, Shareen Forbes

**Affiliations:** ^1^ BHF Centre for Cardiovascular Science, Queens Medical Research Institute University of Edinburgh Edinburgh UK; ^2^ School of Pharmacy, Biodiscovery Institute University of Nottingham, University Park Nottingham UK

**Keywords:** cell therapy, hepatic portal vein, preclinical, ultrasound‐guidance

## Abstract

**Aims:**

Our aim was to determine if ultrasound‐guided HPV injection in mice would provide reproducible and reliable results, as is currently obtained via open laparotomy techniques, and offer a surgical refinement to emulate islet transplantation in humans.

**Methods:**

Fluorescent‐polymer microparticles (20 μm) were injected (27G‐needle) into the HPV via open laparotomy (*n* = 4) or under ultrasound‐guidance (*n* = 4) using an MX550D‐transducer with a Vevo3100‐scanner (FUJIFILM VisualSonics, Inc.). Mice were culled 24‐h post injection; organs were frozen, step sectioned (10 μm‐slices) and 10 sections/mouse (50 μm‐spacing) were quantified for microparticles in the liver and other organs by fluorescent microscopy.

**Results:**

Murine HPV injection, via open laparotomy‐route, resulted in widespread distribution of microparticles in the liver, lungs and spleen; ultrasound‐guided injection resulted in reduced microparticle delivery (*p* < 0.0001) and microparticle clustering in distinct areas of the liver at the site of needle penetration, with very few/no microparticles being seen in lung and spleen tissues, hypothesised to be due to flow into the body cavity: liver median (interquartile range) 4.15 (0.00–4.15) versus 0.00 (0.00–0.00) particle‐count mm^−2^, respectively.

**Conclusions:**

Ultrasound‐guided injection results in microparticle clustering in the liver, with an overall reduction in microparticle number when compared to open laparotomy HPV injection, and high variability in microparticle‐counts detected between mice. Ultrasound‐guided injection is not currently a technique that can replace open laparotomy HPV of islet transplantation in mice.

**What's new**

- In humans, cell and other therapies are transplanted via the hepatic portal vein (HPV) into the liver using ultrasound guidance, whereas in mice an open laparotomy technique is used. We compared the transplantation of fluorescent microparticles via the HPV into the liver in mice using ultrasound guidance versus an open laparotomy technique and found that open laparotomy HPV transplantation was superior.
- In the group of mice that received ultrasound guided HPV transplants, microparticle delivery was not reproducible and did not provide reliable delivery into the liver. In contrast, microparticle delivery via the open laparotomy technique was associated with greater numbers of microparticles detected in the liver with less variation in microparticle numbers between mice in this group.
- Currently, open laparotomy should be continued to be used for hepatic portal vein delivery of cells and therapeutics in mice, until technological advancements in needles and equipment enable techniques that emulate clinical hepatic portal vein injections.

## 1 INTRODUCTION

Cell transplants into the liver have been used in preclinical models and clinical trials in humans for a number of disease indications and with a variety of cell types.^1^, ^2^ Macrophages have been used in phase 1 clinical trials for the treatment of liver cirrhosis,^3^ and islet transplantation to the liver has reached clinical trials with a co‐transplant of Tregs for the treatment of type 1 diabetes.^4^ A common route of administration is via intraportal injection using a catheter or needle.^5^, ^6^

Type 1 diabetes is an autoimmune disease characterised by the destruction of β‐cells in the Islets of Langerhans, situated within the pancreas. Islet transplantation is indicated in people with type 1 diabetes who experience severe hypoglycaemia with impaired awareness of hypoglycaemia.^7^, ^8^ For the process of islet transplantation in humans, isolated human islets are transplanted into the liver of the recipient via laparoscopic guided percutaneous transhepatic portal venous access. This therapy is only used in these select patients due to the requirement of immunosuppression for the lifetime of the graft,^9^, ^10^ and because the graft does not normally result in long‐term insulin independence but typically restores hypoglycaemic awareness. Extensive islet loss secondary to inflammation and autoimmune and alloimmune mechanisms is seen in the first 72 h post‐transplant, often resulting in multiple infusions being required^11^ from multiple different donors—putting extra pressure on this already scarce resource.^10^, ^12^ Islet transplantation needs further refinement and optimisation to ameliorate this islet loss which would subsequently enable more patients to be treated with the islets from donated pancreases.^13^

To study islet transplantation outcomes in humans, preclinical studies in mice are used to test new potential treatments. Currently, preclinical studies are performed using an open laparotomy procedure,^14^ whereby the abdomen of the mouse is opened via a midline incision, the hepatic portal vein (HPV) is located through the temporary relocation of the intestines, and the injection is administered.^14^, ^15^ Although this is a well established preclinical procedure, it does not emulate the procedure used in the clinical setting, where injection into the HPV is done using laparoscopic‐guidance.^10^

Preclinical ultrasound‐guided HPV injections are associated with a diminished risk of bleeding, infection and shorter recovery times versus an open laparotomy technique.

**FIGURE 1:**
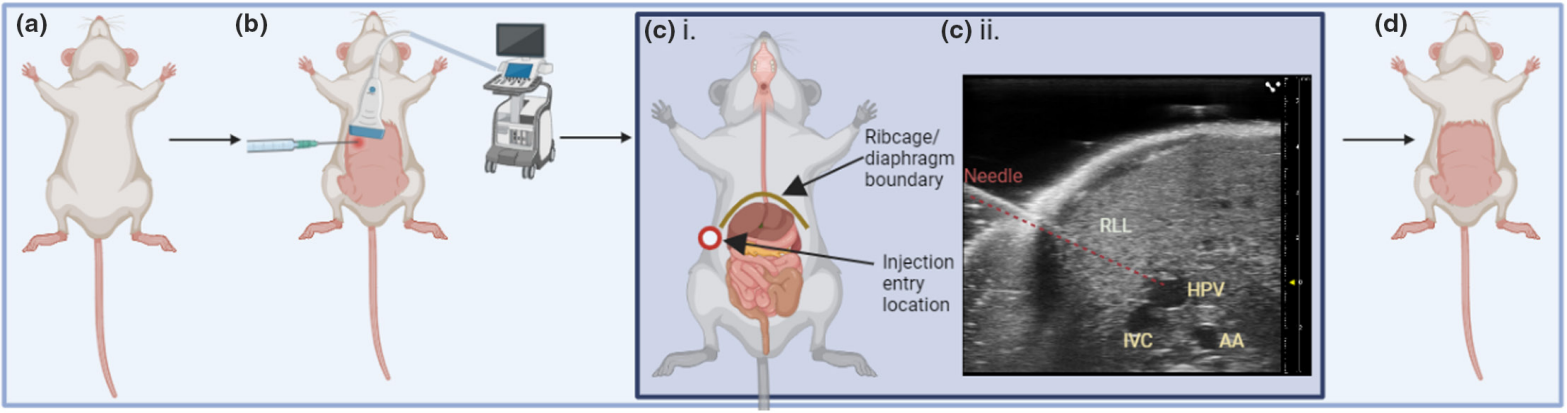
Schematic diagram of ultrasound‐guided trans‐hepatic transverse injection into the HPV via a right abdominal ventral/lateral position; (a) mouse anaesthetised in supine position; (b) ultrasound‐guidance probe used to locate the HPV and align the needle in a transverse plane using a needle rig/rail; (c) (i) the needle passes between the ribcage and intestines—penetration of the intestines would result in mortality, (c) (ii) the needle passes through the skin and RLL of the liver to reach the HPV; (d) needle and ultrasound probe removed, and the mouse is returned to the cage to recover, with no requirement for suturing or skin clips due to the absence of an open wound. AA, abdominal aorta; HPV, hepatic portal vein; IVC, inferior vena cava; RLL, right lateral lobe. Red dashed line, needle passing through skin and RLL.

Little‐to‐no data exists regarding injecting islets or other cells into the HPV via ultrasound‐guidance in preclinical models. Due to ultrasound‐guided injection being less invasive, we hypothesised that this would result in a preclinical surgical procedure refinement.^16^ For the ultrasound‐guided methodology, the needle would pass through the skin and the right lateral liver lobe and enter the HPV, which is visualised using the ultrasound‐guidance probe (Figure 1). As the mouse would not have an open laparotomy incision, this would enable a faster recovery. The aim of this study was to determine if ultrasound‐guided HPV transplantation could be adopted for use in mouse preclinical studies as a reliable and reproducible refinement to current practice and to better emulate the clinical technique.

## 2 METHODOLOGY

### 2.1 Mice

Mouse experiments were conducted under regulated procedures stated on the relevant Home Office licence and all experimental protocols were reviewed and approved by an Institutional Review Board at the University of Edinburgh and were in line with the Animals (Scientific Procedures) Act 1986 Amendment Regulations (SI 2012/3039).

Female NOD.Cg‐Prkdc^SCID^ Il2rg^tm1Wjl^/SzJ (NSG) mice at 18 weeks old (*n* = 4 open laparotomy HPV injection; *n* = 4 ultrasound‐guided HPV injection; weight [mean ± SD] = 23.7 ± 2.0 g) were housed with 12 h light–dark cycles with ad libitum access to food and water. Care was given as per the University of Edinburgh Bioresearch and Veterinary Services guidelines, in conjunction with veterinary support.

Mice were anaesthetised with an induction of 3%–4% isofluorane and maintained on 1%–2% of isofluorane throughout the procedures. Post‐surgery, mice received analgesia with 0.05 mg/kg buprenorphine and 4 mg/kg rimadyl and were administered 0.5 mL saline to help rehydrate, and placed in a warmed cage to recover.

### 2.2 Fluorescent‐polymer microparticles

Microparticles were formed via double emulsion methods from poly (d,l‐lactide‐*co*‐glycolide) (PLGA) with a lactide:glycolide ratio of 85:15 and molecular weight 55 kDa (Evonik) with 10% (w/w) PLGA‐poly(ethylene glycol)‐PLGA (PLGA‐PEG‐PLGA) triblock co‐polymer, synthesised in house as previously described.^17^ Briefly, an aqueous solution of MitoTracker Red (Invitrogen; M7512) was added to a solution of PLGA and PLGA–PEG–PLGA in dichloromethane (DCM). These phases were homogenised for 2 min at 4000 rpm in a Silverson L5M homogeniser (Silverson Machines) to form the water‐in‐oil emulsion. This primary emulsion was transferred to 200 mL 0.3% (w/v) poly(vinyl alcohol) solution and was homogenised for a second time at 9000 rpm for 2 min. The resultant double emulsion was stirred at 300 rpm on a Variomag 15‐way magnetic stirrer for 4 h to facilitate DCM evaporation. Microparticles were then washed and lyophilised (Edwards Modulyo; IMA Edwards) until dry. One gram total polymer was used to fabricate 20 μm microparticles with 200 μg of encapsulated MitoTracker Red.^17^ A dose of 0.1 mg of microparticles (estimated via a haemocytometer to be approximately 7000 microparticles) was suspended in sterile saline (200 μL) before immediate injection into mice. The fluorescent microparticle formulation enabled an assessment of the success of the injection to be determined via the detection of microparticles in the liver and other organs on histological analysis.

### 2.3 Open laparotomy mouse studies

Anaesthetised NSG mice (*n* = 4) were opened at the abdomen with a midline incision. The intestines were temporarily relocated onto a saline soaked swab to the side of the incision, and the HPV located.

The injection was performed into the HPV using a 1 mL syringe and adapted 27G needle (with 45% shorter bevel length). Haemostatic agent was applied to the venous injection site once the needle was removed. The mice were sutured and skin clipped, as previously described.^18^

### 2.4 Ultrasound‐guided mouse studies

Anaesthetised NSG mice (*n* = 4) were placed onto a heated scanning table for ultrasound‐guided injections. The HPV was located in a transverse plane using the ultrasound probe (MX550D‐transducer with a Vevo3100‐scanner; FUJIFILM VisualSonics, Inc.) and a 1 mL Hamilton syringe and adapted 27G needle were aligned to pass through the liver and into the HPV.

### 2.5 Mouse culls and tissue collection

Mice were culled 24 h post‐transplant via cervical dislocation, and severing of a main blood vessel for confirmation.

Liver, lung and spleen tissues were collected from all mice, placed on dry ice immediately, then stored at −80°C.

### 2.6 Histological tissue staining, imaging and microparticle quantification

Liver, lung and spleen tissues were set into optimal cutting temperature (Epredia™ Cryomatrix™; 6769006) compound and stored at −80°C.

Tissues were cryosectioned (10 μm step sections) at −20°C. Serial sections were collected, stained with 4′,6‐diamidino‐2‐phenylindole (DAPI; Invitrogen; D1306) and quantified.

For quantification of the mouse livers, 60 serial sections were collected per mouse (30 slides, 2 sections per slide), every fifth slide (slides 5, 10, 15, 20, 25) stained with DAPI (Invitrogen; D1306) and microparticles quantified by counting numbers in standardised area (five 0.244 mm^2^ boxes drawn randomly on each of the 10 tissue sections per mouse).

For lung and spleen tissue analysis, 20 serial sections were collected per tissue per mouse (10 slides, 2 sections per slide); slides 3 and 6 were then analysed per tissue. Ten 0.244 mm^2^ boxes were drawn across the four sections of lung or spleen tissues per mouse for quantification of distribution of microparticles throughout the different tissues.

Imaging was performed on a Zeiss Axioscanner for whole slide imaging at excitation wavelengths of 353 nm (DAPI) and 548 nm (microparticles). A 0.244 mm^2^ box was used to randomly sample each of the tissue sections for the four open laparotomy and four ultrasound‐guided mice.

### 2.7 Statistical analysis

Data are represented as percentage frequency of microparticles per 0.244 mm^2^ analysis area.

Statistical analysis was performed using a chi‐squared test to examine differences between the distribution of microparticles throughout liver, lung and spleen tissue when using open laparotomy versus ultrasound‐guided HPV injection.

## 3 RESULTS

### 3.1 Quantitative analysis shows greater numbers of microparticles that distribute widely and more evenly throughout the liver following open laparotomy versus ultrasound‐guided HPV injection in mice

Microparticles were widely distributed in the liver following open laparotomy HPV injection versus ultrasound‐guided HPV injection which showed a limited and heavily skewed distribution, skewness: 1.4 versus 6.9, respectively. Microparticle counts were greater following open laparotomy HPV versus ultrasound‐guided injection: median (interquartile range) 4.15 (0.00–4.15) versus 0.00 (0.00–0.00) particle‐count mm^−2^, respectively (*p* < 0.0001).

### 3.2 Qualitative analysis of microparticles in liver tissue reveals singular ‘ring’ of high microparticle density following ultrasound‐guided injection

**FIGURE 2:**
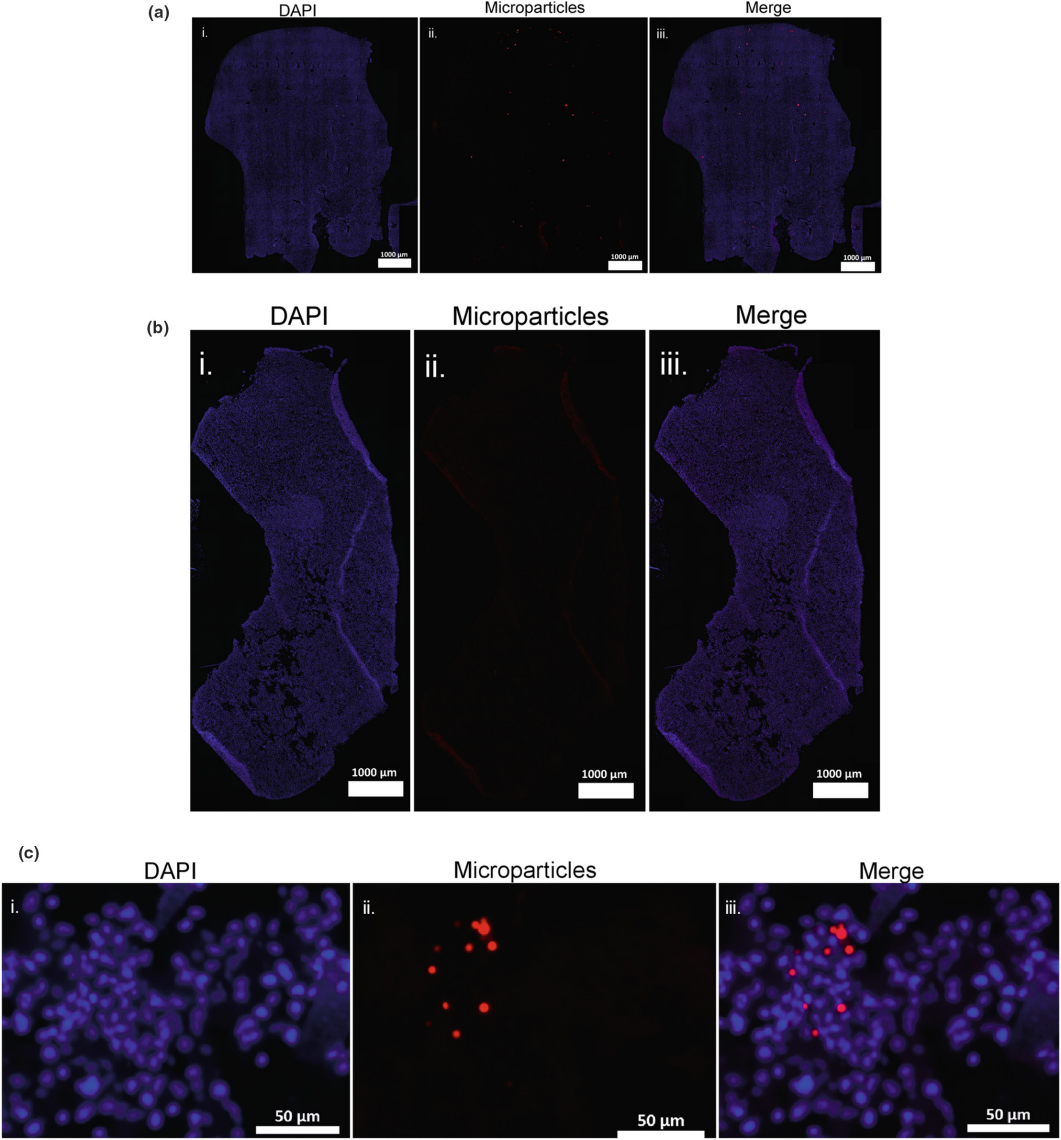
(a) Representative image of microparticle distribution from open laparotomy HPV injection in mouse liver tissue. Scale bar 1 mm. (b) Representative image of microparticle distribution from ultrasound‐guided HPV injection in mouse liver tissue. Scale bar 1 mm. (c) Example of microparticle ‘ring’ in mouse liver tissue following ultrasound‐guided injection of the HPV. Scale bar = 50 μm. (i) Blue = DAPI; (ii) red = PLGA/PLGA‐PEG‐PLGA‐MitoTracker Red 20 μm microparticles; (iii) merged channels. DAPI, 4′,6‐diamidino‐2‐phenylindole; HPV, hepatic portal vein; PEG, polyethylene glycol; PLGA, poly (d,l‐lactide‐*co*‐glycolide).

Qualitative analysis showed an even distribution of microparticles throughout liver tissue following open laparotomy injection (Figure 2a); whereas following ultrasound‐guided injection there were typically no microparticles detected (Figure 2b), other than a single random patch/‘ring’ of microparticle clustering (Figure 2c) seen.

### 3.3 Microparticles enter systemic circulation in mice when injected via open laparotomy techniques, as seen in lung and spleen analysis

**FIGURE 3:**
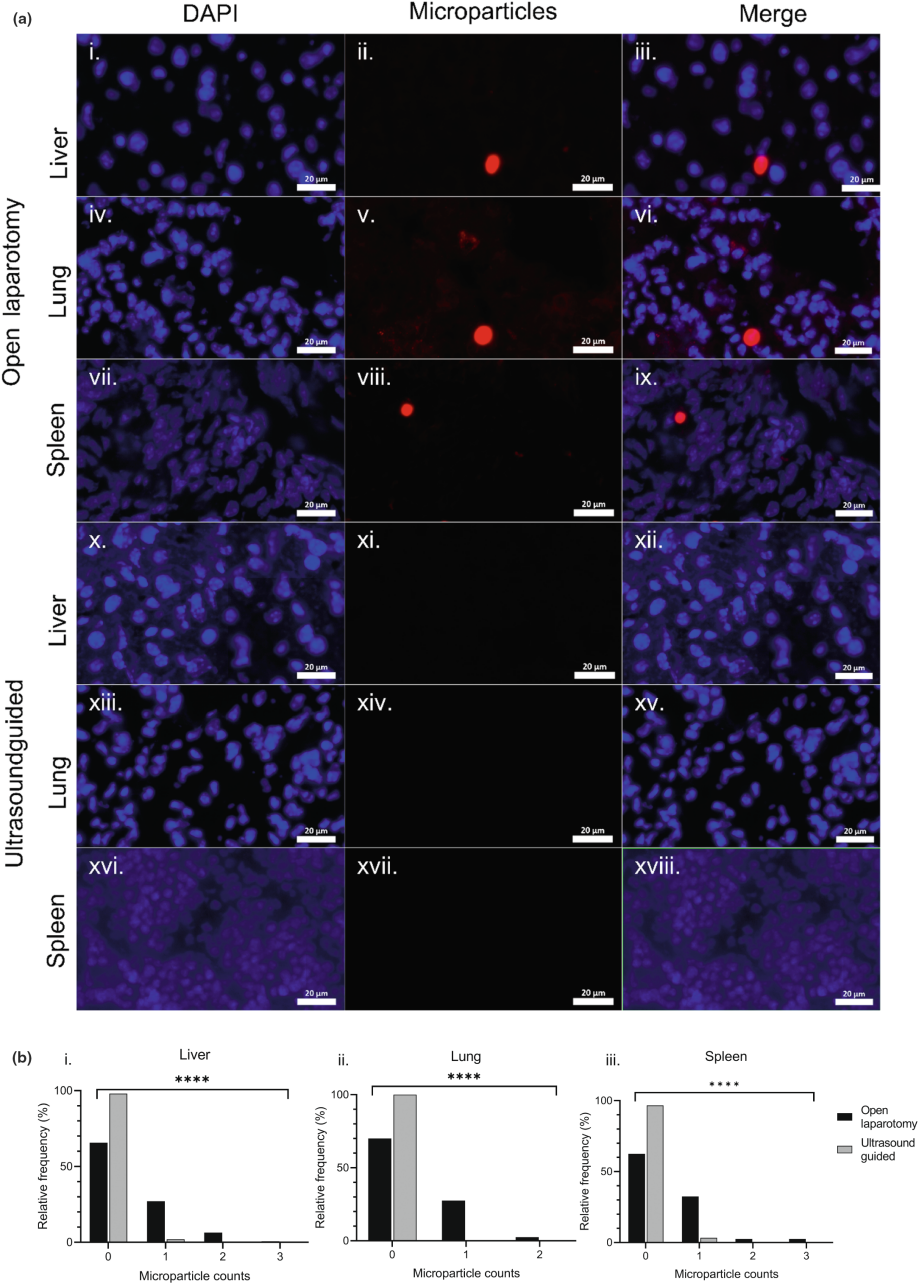
(a) Representative images from microparticle quantification in female NSG mice following open laparotomy or ultrasound‐guided HPV injection. Open laparotomy: (i) liver tissue DAPI; (ii) liver tissue microparticles; (iii) liver tissue merge; (iv) lung tissue DAPI; (v) lung tissue microparticles; (vi) lung tissue merge; (vii) spleen tissue DAPI; (viii) spleen tissue microparticles; (ix) spleen tissue merge. Ultrasound guided; (x) liver tissue DAPI; (xi) liver tissue microparticles; (xii) liver tissue merge; (xiii) lung tissue DAPI; (xiv) lung tissue microparticles; (xv) lung tissue merge; (xvi) spleen tissue DAPI; (xvii) spleen tissue microparticles; (xviii) spleen tissue merge. Blue—DAPI; red—20 μm PLGA/PLGA‐PEG‐PLGA‐MitoTracker Red microparticle. Scale bar—20 μm. (b) Frequency distributions of microparticles analysed throughout (i) liver, (ii) lung and (iii) spleen tissues following open laparotomy (*n* = 4) or ultrasound‐guided (*n* = 4) HPV injection in NSG female mice. **** *p* < 0.0001; Chi‐squared statistical analyses. DAPI, 4′,6‐diamidino‐2‐phenylindole; HPV, hepatic portal vein; NSG, NOD.Cg‐Prkdc^SCID^ Il2rg^tm1Wjl^/SzJ; PEG, polyethylene glycol; PLGA, poly (d,l‐lactide‐*co*‐glycolide).

Following open laparotomy HPV injection, in addition to microparticles being located in the liver, microparticles were also detected in the lung and spleen tissue; in contrast following ultrasound‐guided injection, no microparticles were detected in the lung tissue and only one microparticle was seen in the spleen (Figure 3). Overall, there were greater numbers of microparticles following open laparotomy injection versus ultrasound‐guided injection in liver, lung and spleen tissue (all *p* < 0.0001; Figure 3b).

## 4 DISCUSSION

Our study shows that ultrasound‐guided HPV injection of fluorescent 20 μm PLGA with 10% PLGA‐PEG‐PLGA microparticles in mice results in reduced delivery and significantly skewed distribution of microparticles throughout the liver. In contrast, delivery of microparticles by the open laparotomy route resulted in greater numbers of particles delivered to the liver with a more even distribution throughout the whole liver. Clinically when cell therapies are delivered to the liver via the portal circulation a proportion will enter the systemic circulation via the vena cava and then lodge in the pulmonary and splenic circulation.^19^ In our study, analysis of lung and spleen tissue shows that microparticles are detected in these tissues following open laparotomy HPV injection.

We have previously shown that following open laparotomy HPV injection, 20 μm PLGA‐MitoTracker microparticles are delivered in high numbers to the liver and then enter the systemic circulation and are detected in the lung and spleen.^18^, ^20^

Interestingly, when using the ultrasound‐guided technique very few microparticles were seen to be dosed to the liver, and only a single microparticle was seen in the lung and spleen tissue. This strongly suggests that microparticles did not enter the portal circulation to then be introduced into the systemic circulation. The minimal numbers of microparticles in the liver indicate an unsuccessful injection.

Our study was set up to emulate islet transplantation. Islets are clusters of cells and mouse islets have mean diameter of 100 μm ranging between 50 and 200 μm. A 27G needle with an inner diameter of 210 μm is required to inject islets into the HPV. We therefore used 27G needles in this study following analysis to ensure that minimal islet fragmentation was observed and with accurate islet dosing. It was noted in pilot studies that a standard bevel length failed to fully fit inside the portal vein at the injection angle that was required under ultrasound guidance. Therefore, in order to better access the HPV, we customised the needles to have a 45% reduction in bevel length, however, this adaptation did not improve quantities of microparticles detected in the liver. Microparticles could have flowed into the body cavity instead of being fully injected into the HPV. This hypothesis was further supported with visualisation observed on the ultrasound screen of back flow at the commencement of the injection that is, flow from the needle back into the body cavity, away from the vessel. Furthermore, clustering of microparticles at the very edge of liver tissues was observed during image analysis, reinforcing the hypothesis that microparticles were present in the body cavity. Although this surgery was performed by a skilled ultrasound operator with >10 years' experience of preclinical ultrasound scanning,^21^ it was difficult to ascertain at the time of injection whether an injection was fully successful (i.e. whether all of the microparticles suspended in solution entered into the HPV); open laparotomy HPV injection provides a more robust indication of success at the time of injection due to the clear visualisation of the needle entering the vessel and being within the vessel upon commencement of the injection.

A caveat of using these fluorescent microparticles is their smaller diameter versus islets.^22^ However, mouse islets are more difficult to detect once transplanted into the liver, hindering rapid analysis to compare ultrasound guided and open laparotomy techniques. The model that was used here usefully reflects cell delivery at the site of injection but is limited to delivery analysis only, and so the data cannot be used to draw inferences on cell survival. Microparticles also do not possess the elasticity which is seen with the cell membranes in islets, and, therefore, are structurally more rigid when flowing through the vasculature, hence why the chosen microparticle size used was not the exact size of islets. Larger microparticles and higher doses of microparticles result in extensive necrosis within the liver, and, therefore, this could further hinder analysis and accurate quantification, hence why the dose of 0.1 mg, which has also been validated in open laparotomy techniques to have a high distribution in liver tissue, was chosen here.

Ultrasound‐guided injection into other blood vessels and organs for the administration of various cell types in preclinical studies are well reported.^1^, ^2^, ^23^ The requirement for a 27G needle to allow for the islets to be transplanted is, in this scenario, the main confounding factor as to why this method is not working adequately, and results in the bevel of the needle not fully entering the HPV for reliable dosing in the mouse.

Although ultrasound‐guided injections in mice were not successful, fluoroscopic imaging holds potential and has been used in smaller human vessels successfully^24^ and, therefore, in preclinical mouse studies this may be a future technique to adopt to assist with injection guidance. Further needle adaptations to allow for the 27G diameter but with a shorter bevel than attempted here, future technological advancements in ultrasound‐guided equipment, or investigation into the use of ultrasound‐guided HPV injection in larger animal models including rats could allow for this technique to be pursued in the future.

## 5 CONCLUSIONS

The reduced delivery and distribution of microparticles by ultrasound‐guided injections in liver tissues when compared to open laparotomy HPV injection, suggests that ultrasound‐guided injections into the HPV were not successfully achieved in this mouse model. Ultrasound‐guided injections have been shown to be useful in other applications,^25^ however, currently ultrasound‐guided injection of the HPV for islet transplantation in mice is not a reliable method to emulate clinical islet transplantation in humans.

## AUTHOR CONTRIBUTIONS

Sophie L. Walker performed the experimental animal culls, tissue preparation, laboratory testing, data analysis and manuscript writing, revising and editing. June Noble performed the open laparotomy surgical techniques and animal husbandry. Adrian Thomson performed the ultrasound‐guided surgical techniques. Carmel M. Moran assisted with data interpretation and manuscript revisions. David Mellis participated in study design and data interpretation. I‐Ning Lee and Lisa J. White provided the microparticles used for these studies and assisted with manuscript revisions. Shareen Forbes participated in study design data interpretation and manuscript writing and editing. Shareen Forbes is the guarantor of this work and as such had full access to all the data in this study and takes responsibility for the integrity of the data and the accuracy of the data analysis. All authors approved the manuscript for publication.

## FUNDING INFORMATION

S.L.W. is supported by a 4‐year BHF PhD studentship (FS/19/55/34890). The work was also supported by a UK Regenerative Medicine Platform Grant: MR/K026666/1; MRC Regenerative Medicine Grant: MR/S03692X/1; Acellular/Smart Materials—3D Architecture: UK RMP Hub grant: MR/R015651/1; and an Edinburgh and Lothian Health Foundation Award.

## CONFLICT OF INTEREST STATEMENT

S.F. collaborates with, and receives funding from, Novo Nordisk, Cell Therapy Programme, Copenhagen, Denmark. S.L.W. presented this work as part of the Basic Science Poster Award poster presentations at the Diabetes UK Professional Conference in Liverpool, UK (26–28th April 2023).

## Data Availability

The data that support the findings of this study are available from the corresponding author upon reasonable request.
